# Infantile-Onset Charcot–Marie–Tooth Disease With Pyramidal Features and White Matter Abnormalities Due to a *De novo MORC2* Gene Variant: A Case Report and Brief Review of the Literature

**DOI:** 10.3389/fneur.2021.718808

**Published:** 2021-09-22

**Authors:** Ivana Frongia, Susanna Rizzi, Margherita Baga, Laura Maria Ceteroni, Carlotta Spagnoli, Grazia Gabriella Salerno, Daniele Frattini, Milja Kaare, Francesco Pisani, Carlo Fusco

**Affiliations:** ^1^Struttura Complessa di Neuropsichiatria Infantile, Dipartimento Materno-Infantile, Azienda Unità Sanitaria Locale - Istituto di Ricerca e Cura a Carattere Scientifico di Reggio Emilia, Reggio Emilia, Italy; ^2^Blueprint Genetics, Esopoo, Finland; ^3^Child Neuropsychiatry Unit, Medicine and Surgery Department, Neuroscience Section, University of Parma, Parma, Italy

**Keywords:** Charcot–Marie–Tooth, axonal neuropathy, pyramidal signs, white matter abnormalities, *MORC2* gene

## Abstract

**Background:** Charcot–Marie–Tooth (CMT) is the most frequent group of inherited neuropathies and includes several heterogeneous phenotypes. Over 80 causative genes have been described so far. Variants in the microrchidia family CW-type zinc finger 2 (*MORC2*) gene have been described in several axonal polyneuropathy (CMT2) patients with childhood or adult onset. Occasionally more complex phenotypes with delayed milestones, severe hypotonia, intellectual disability, dystonic postures, pyramidal signs, and neuroimaging abnormalities have been reported.

**Case Presentation:** We report on a patient with a *de novo MORC2* gene variant (c.1181A>G p.Tyr394Cys) with a history of developmental delay, axial hypotonia, progressive gait disorder with dystonic features, and intentional tremor. At the age of 8 years, he showed bilateral pyramidal signs (clonus, increased tendon reflexes, and Babinski sign) and bilateral pes cavus. The first neuroimaging performed at the age of 3 years demonstrated white matter abnormalities in the posterior periventricular zone, in the frontal lobes bilaterally and at the midbrain, stable during childhood and adolescence. Nerve conduction studies (NCS) were negative until the age of 15 years, when a sensory axonal neuropathy appeared. The association between pyramidal signs and neuropathy due to the *MORC2* gene variant is increasingly being highlighted, although a neuroradiological correlate is evident only in about half of the cases. Longitudinal nerve conduction velocity (NCV) are helpful to identify late-onset features and provide useful information for diagnosis in patients with rare neurogenetic disorders.

**Conclusions:** Characterization of complex neurological disorders is important to delineate the expanding phenotypic spectrum of *MORC2*-related disease, to confirm if possible the pathogenicity of the variants and to deepen the genotype–phenotype correlation.

## Introduction

CMT disease is the most frequent inherited neuropathy, with an estimated prevalence in Europe of 10–28:100,000 (1). Over 80 causative genes have been described so far. Variants of the gene microrchidia family CW-type zinc finger 2 (*MORC2*) have been described in several axonal CMT patients, typically characterized by childhood or early adulthood onset of progressive weakness and sensory impairment. Occasionally delayed milestones, intellectual disability, dystonic attitude, pyramidal signs and brain magnetic resonance imaging (MRI) abnormalities have been reported (2). Here we report a patient with a *de novo MORC2* gene variant highlighting the neurophysiological and neuroradiological findings.

## Case Report

Our patient is a 15-year-old boy with motor development characterized by gross motor delay, independent sitting at 9 months, and independent walking at 19 months. Language development was also reported as delayed; however, a formal neuropsychological profile is unavailable. No facial dysmorphism was observed. Transient internal rotation on the left superior limb was first observed at the age of 2 years and then disappeared. Subsequently, a complex neurological disorder became evident, with mild axial hypotonia, gait disorder, and intentional tremor. At the age of 8 years, bilateral pyramidal signs (unstained plantar clonus, increased tendon reflexes, and Babinski signs) appeared. His last neurological examination at 15 years of age showed asymmetrical distal dystonic posturing, spasticity, and bilateral pes cavus. Other neurological signs presented a stable course. Owing to dystonic features, levodopa and carbidopa therapy was set up, but it was withdrawn due to ineffectiveness. He followed an individualized physiotherapy program.

**Figure 1:**
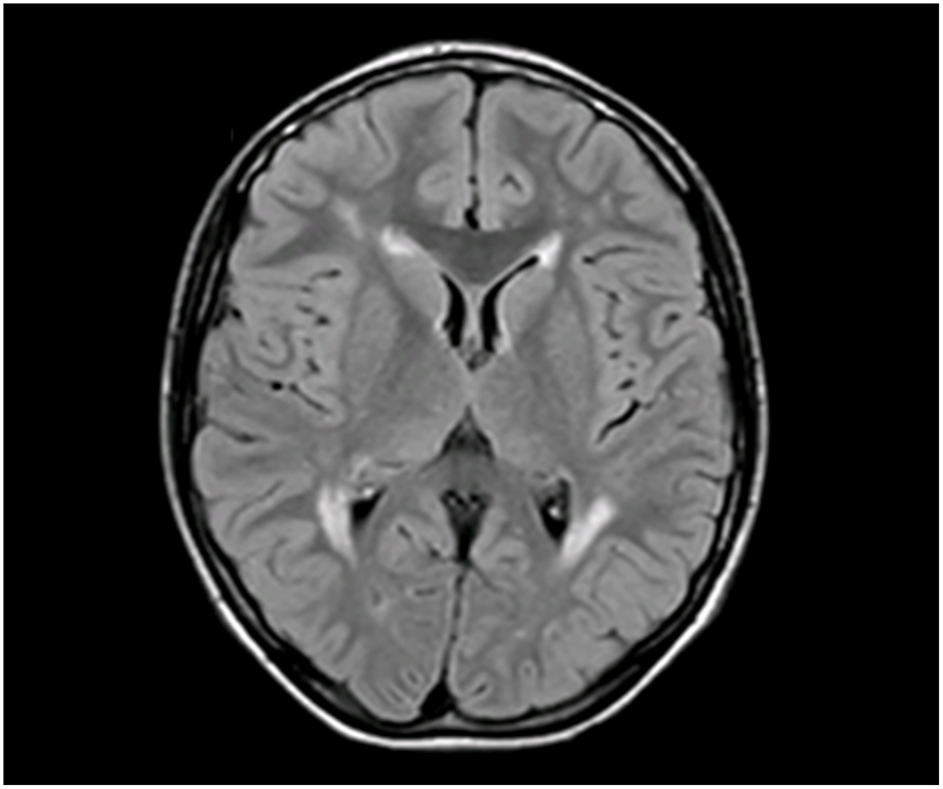
Brain MRI at 3 years, axial view: supratentorial white matter increased the T2 signal in the periventricular and frontal areas.

First brain MRI (1 Tesla), at the age of 3 years, demonstrated areas of white matter hypomyelinations stable during childhood and adolescence (Figure 1).

Nerve conduction studies (NCS) and electromyography (EMG) were performed at 4, 7, and 15 years. EMG was performed with a four-channel EMG machine (EBNeuro, Galileo NT PMS 2001, Mito II; Florence, Italy). EMG activity was amplified, and the filter band was set at the lower filter of 10 Hz to the upper filter of 10 kHz with a sweep duration of 100 ms. Sensitivity was assessed according to the potential amplitude varying between 100 and 500 mV. Standard techniques of electrophysiological studies were used for motor and sensory conduction velocities and amplitude measurements of the median, ulnar, peroneal, tibial, and sural nerves. Needle EMG of the bilateral anterior tibial, extensor indicis, and gastrocnemius muscles was also performed. The first two evaluations showed normal values. The last examination at the age of 15 years revealed axonal sensory polyneuropathy in all four limbs (Table 1). The patient has undergone serial diagnostic investigations, all unremarkable (Table 2). Considering the persistence of a complex neurological phenotype and assuming its genetic origin, whole-exome sequencing (WES) was performed including detection for both sequence variants (with mean coverage of 165× and 99.5% of the target nucleotides had a 0.20× read depth) and CNVs (sensitivity of 92% at one exon level) [Talevich E, Shain AH, Botton T, Bastian B. CCNV kit: genome-wide copy number detection and visualization from targeted DNA sequencing. *PLoS Comput Biol*. (2016) 12:e1004873] (https://blueprintgenetics.com/tests/whole-exomesequencing/whole-exome-family/). Informed consent was from parents obtained in accordance with international ethical standards. A heterozygous missense *de novo* variant c.1181A>G, p.(Tyr394Cys) in the *MORC2* gene was identified. Mutation nomenclature is based on GenBank accession NM_001303256.2. This variant is absent in the large reference population cohorts of the Genome Aggregation Database (gnomAD, *n* > 120,000 exomes and >15,000 genomes). The variant is predicted as damaging by used *in silico* tools SIFT, PolyPhen, and MutationTaster. The variant was initially classified as a variant of uncertain significance (VUS), as there were not enough evidence to completely support its pathogenicity. Parental testing and recent publications identifying the same variant in other patients allowed reclassification of the variant to pathogenic. No other variants in known disease-causing genes were identified. Whole-exome data of the patient were also analyzed for secondary findings, according to recommendations of the American College of Medical Genetics and Genomics (ACMG; PMID 27854360); analysis was negative.

**Table 1:** Nerve conduction study performed at the age of 15 years.

**Sensory nerve conduction study**					
	**Latency** **(ms)**	**Amplitude** **(μV)**	**Duration** **(ms)**	**Area** **(μVms)**	**VCN** **(m/s)**
**Upper limbs**					
Median nerve (R)	2.1	7.16 (>20)	0.8	12.2	51.5
Median nerve (L)	2.5	9.39 (>20)	1	5.98	46.5
Ulnar nerve (R)	2.5	2 (>15)	0.5	3.79	43
Ulnar nerve (L)	2.5	1.50 (>15)	0.7	3.79	44.5
**Lower limbs**					
Sural nerve	SAP not evocable bilaterally				

*R, right; L, left; VC, conduction velocity; SAP, sensory action potential. Results in parentheses are normal values*.

**Table 2:** Diagnostic investigations performed and resulted negative.

**Diagnostic investigations performed**	
Metabolic exams	Urinary organic acids, plasma amino acids profile, vitamin E, lactate, plasma very long-chain fatty acids (VLCFA), and lysosomal enzymes
Genetic exams	Array-CGH, fragile-X, FISH22 and direct sequencing of *DYT1, DYT5, DYT11*, connexin 47, *SCL2A1* and *EGR2*, next-generation sequencing (NGS) panel evaluating genes associated with hypomyelinating leukodystrophies, NGS panel evaluating genes involved in hereditary spastic paraplegias (HSP)
Instrumental exam	Electroencephalograms
Others	Eye examination, cerebrospinal fluid analysis

## Discussion

The *MORC2* gene (OMIM 616661) encodes a member of the 92 MORC protein superfamily, a nuclear protein characterized by an N-terminal ATPase domain, a central zinc-finger CW domain, and a divergent C-terminal region with one or more coiled coils (2). It is involved in biological functions, such as regulation of heterochromatin condensation in DNA damage, transcriptional repression, lipid metabolism, cytoskeleton functions, and axonal transport (3, 4). Several studies pointed out the role and expression of MORC2 in both central and peripheral nervous systems (2, 5–7).

Up to now, a broad phenotypic spectrum associated with MORC2-related disease has been delineated. Primarily autosomal dominant CMT disease axonal type 2Z (OMIM 616688) with possible central nervous system involvement was described. Subsequently, both early-onset patients with severe and progressive SMA-like features and late-onset cases (usually less severe, with distal muscle weakness, and sensory impairment) have been reported (2, 7, 8). Recently, more complex *MORC2*-related clinical and neuroradiological pictures such as Leigh-like syndrome have been described. Microcephaly, seizures, cognitive impairment, pigmentary retinopathy, hearing loss, and diaphragmatic paralysis can be sometimes associated (2, 7–9). We report a patient with progressive neurological phenotype characterized by both peripheral and central nervous system (PNS, CNS) involvement due to a *de novo* MORC2 gene variant with stable neuroradiological picture and evolutive neurophysiological findings.

Typically, *MORC2* variants cause early-onset length-dependent sensory-dominant axonal neuropathy. Rarely, some patients show normal NCV: they are often pediatric or young adult subjects, but no further information is available about the follow-up (10, 11). Our patient performed serial NCS: NCV was negative until the age of 15 when sensory axonal neuropathy appeared. According to our experience, instrumental evidence of neuropathy may appear later than the onset of the clinical picture.

Recent studies reveal that the *MORC2* gene is expressed in both human and murine neural tissues, (neurons, axons, and Schwann cells) (5). Its role in the development, maturity, and regulation in PNS and CNS has been demonstrated in animal models. *MORC2* dysfunction led to axonal swellings and altered expression of different neurotransmitter receptors (5). Brain MRI is often normal, but in recent years reports of brain abnormalities *MORC2*-related have been increasing such as cerebellar atrophy and white matter abnormalities (2, 7, 10–13). Finally, only a single case with cortical dysplasia has been described (10) and rarely Leigh-like neuroradiological features with bilateral symmetric abnormalities in the basal ganglia, brainstem, and/or cerebellum were detected (10). Of note, longitudinal neuroradiological studies are still limited but in our experience, white matter changes remain stable at subsequent controls performed during adolescence.

The c.1181A>G, p.(Tyr394Cys) variant in *MORC2* has been identified in literature in other three patients with axonal sensory polyneuropathy and white matter abnormalities. Including our patient, pyramidal signs are present in 50% of individuals (Table 3).

**Table 3:** Clinical, neuroradiological, and neurophysiological features of individuals with the p.(Tyr394Cys) variant on the MORC2 gene.

**Affected individuals (sex)**	**MORC2 gene variant**	**Pyramidal signs**	**Brain MRI**	**Type of neuropathy**
**Ando et al**. **(****7****)** **(M)**	c.1181A>G p.Tyr394Cys	None	Not available	Axonal sensory polyneuropathy
**Guillen Sacoto et al**. **(****10****)** **(F)**	c.1181A>G p.Tyr394Cys	Spasticity	Multiple foci of chronic hemosiderin deposition within the supratentorial and infratentorial white matter	Axonal sensory polyneuropathy
**Guillen Sacoto et al**. **(****10****)** **(M)**	c.1181A>G p.Tyr394Cys	None	Generalized brain atrophy and scattered subcortical and deep white matter microangiopathic changes	Axonal sensory polyneuropathy
**Our patient (M)**	c.1181A>G p.Tyr394Cys	Unsustained plantar clonus, increased tendon reflexes, Babinski signs	Hypomyelination with areas of impaired white matter signal in the posterior periventricular site (right > left), bilateral frontal level and midbrain, stable during childhood and adolescence	Axonal sensory polyneuropathy

*M, male; F, female*.

The p.(Tyr394Cys) variant has also been identified in four individuals reported in ClinVar (ClinVar ID 432089), where its interpretation of pathogenicity is conflicting. However, based on the patients reported until now, the *de novo* occurrence of the variant, the absence of the variant in control population databases, and the damaging prediction of *in silico* tools used, we believe that the p.(Tyr394Cys) variant is to be considered as pathogenic. HGMD Professional (version 2021.1) lists altogether 25 variants in *MORC2* in association with *MORC2*-related phenotypes. All of the reported variants are missense variants. The reported variants are located along the entire gene, and at the moment there seems to be no clear correlation between variant location and clinical phenotype caused by the variant. Guillen Sacoto et al. (10) recently highlighted a correlation between the degree of HUSH hyperactivation in cellular assays and the severity of central nervous system involvement. The variability of the genotype–phenotype correlation probably reflects the limitations of our understanding of the relationship between the biologic functions of the affected protein, the postulated modulator genes, and environmental factors.

In conclusion, the emerging phenotype of *MORC2*-related pathologies is not limited to the symptoms of polyneuropathy but extended through the involvement of the CNS. The association with pyramidal signs concomitant with neuropathy is increasingly being highlighted (14, 15), although a neuroradiological correlate is evident only in about half of the cases. Currently, pyramidal signs are reported in a minority of *MORC2*-patients; collecting a larger case study could define which variants are most associated with this feature (2). Longitudinal NCV is helpful in identifying late-onset features and providing useful information for the diagnosis and long-term follow-up of patients with rare progressive neurogenic disorders and previously normal NCV. Characterization of complex cases is important in delineating the expanding phenotype spectrum of *MORC2*-related clinical pictures and, if possible, in confirming the pathogenicity of the variants found and better deepening the genotype–phenotype correlation.

## Data Availability Statement

The datasets presented in this study can be found in online repositories. The names of the repository and accession number can be found at: https://www.ncbi.nlm.nih.gov/genbank/ (NM_001303256.2).

## Author Contributions

IF, MB, LMC, and FP contributed to manuscript development. SR, CS, GGS, DF, and CF contributed to manuscript development, patient management, and diagnostic definition. MK contributed to diagnostic definition. All authors contributed to the article and approved the submitted version.

## Conflict of Interest

MK was employed by company Blueprint Genetics. The remaining authors declare that the research was conducted in the absence of any commercial or financial relationships that could be construed as a potential conflict of interest.

## Publisher's Note

All claims expressed in this article are solely those of the authors and do not necessarily represent those of their affiliated organizations, or those of the publisher, the editors and the reviewers. Any product that may be evaluated in this article, or claim that may be made by its manufacturer, is not guaranteed or endorsed by the publisher.
